# Supplementary material for: The Phosphorylation Status of Drp1-Ser637 by PKA in Mitochondrial Fission Modulates Mitophagy via PINK1/Parkin to Exert Multipolar Spindles Assembly during Mitosis
Source: Biomolecules. 2021 Mar 13;11(3):424. doi: 10.3390/biom11030424 (PMC7998912; doi:10.3390/biom11030424)
Supplement: Supplementary file 1 [file biomolecules-11-00424-s001.pdf]

biomolecules

MDPI

Supplementary Materials

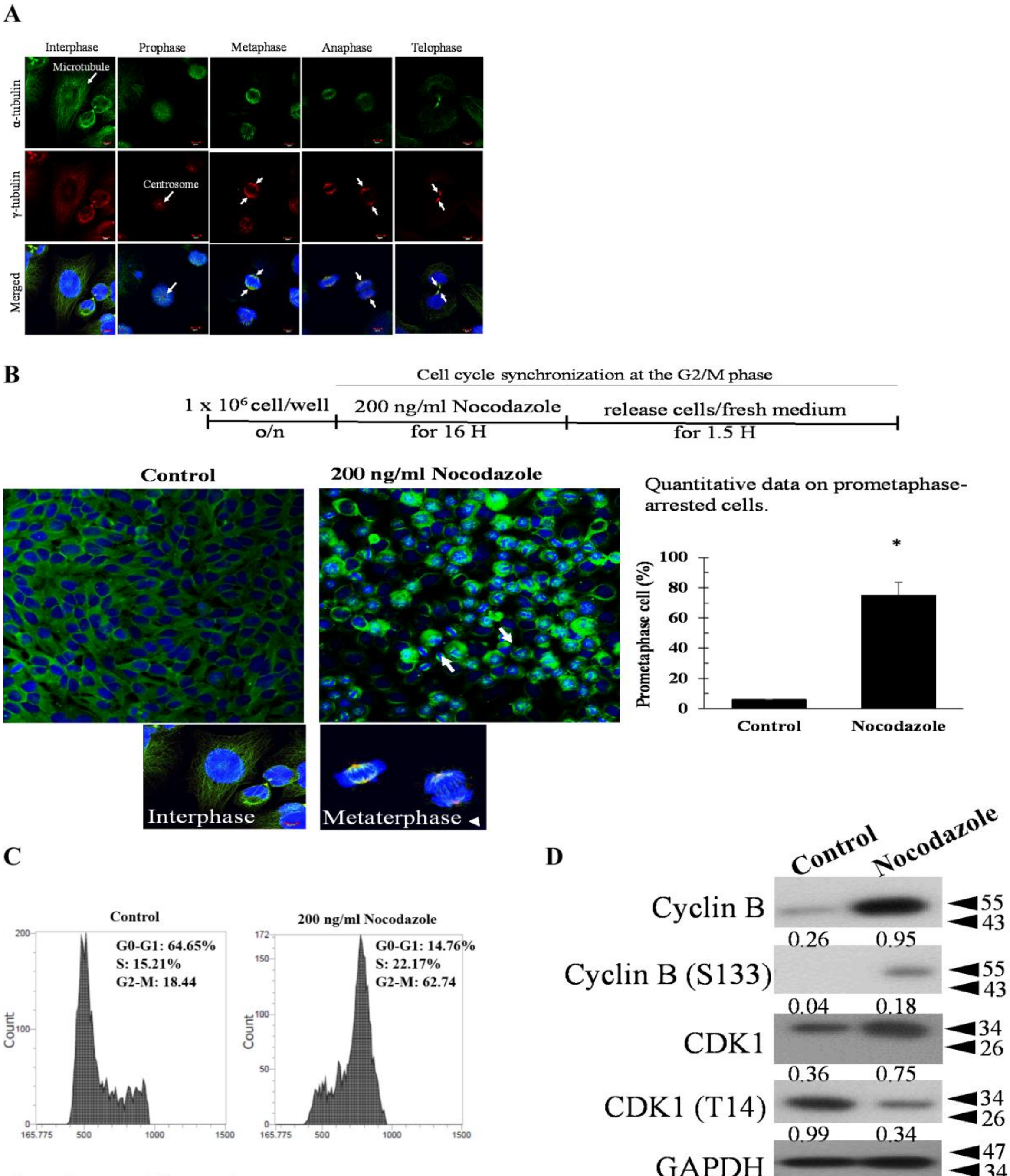

Supplement Figure 1

Supplementary Figure 1. Nocodazole was used to synchronize the cell division cycle. HeLa cells were stained with anti-α-tubulin antibody (green, microtubule), anti-γ-tubulin antibody (red, Centrosome), and DAPI (blue, DNA). Scale bars, 5 μm. Following treatment for 16 hrs with or without 200 ng/ml nocodazole, HeLa cells were observed under a microscope (magnification, ×20).

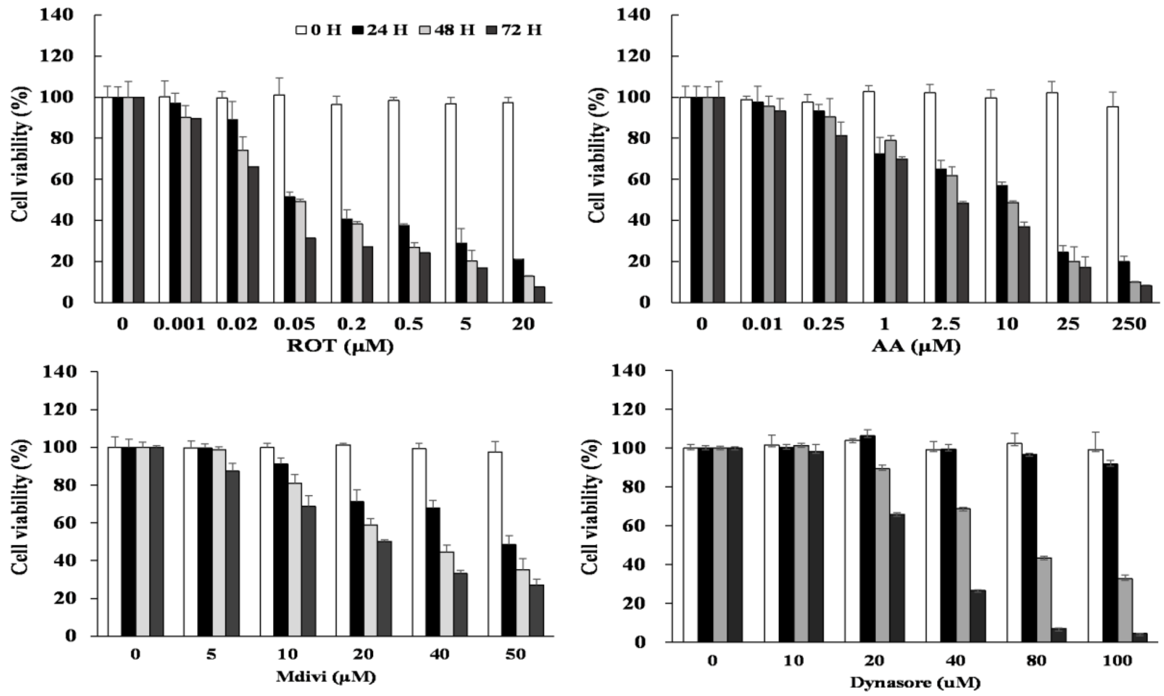

	IC_{50} (µM)			
(Hours)	ROT	AA	Mdivi-1	Dynasore
24	0.19 ± 0.25	11.87 ± 1.63	48.3 ± 3.74	>100
48	0.11 ± 0.06	8.98 ± 0.63	36.24 ± 2.13	81.24 ± 7.39
72	0.04 ± 0.02	4.99 ± 0.86	22.27 ± 3.41	30.21 ± 4.64

Supplement Figure 2

Supplementary Figure 2. Decreased growth rate is associated with mitochondrial ETC blockade in HeLa cells. Growth rate of HeLa cells in the presence of m mitochondrial ETC complex inhibitor, ROT or AA, and dynamin inhibitor Mdivi-1 or Dynasore.

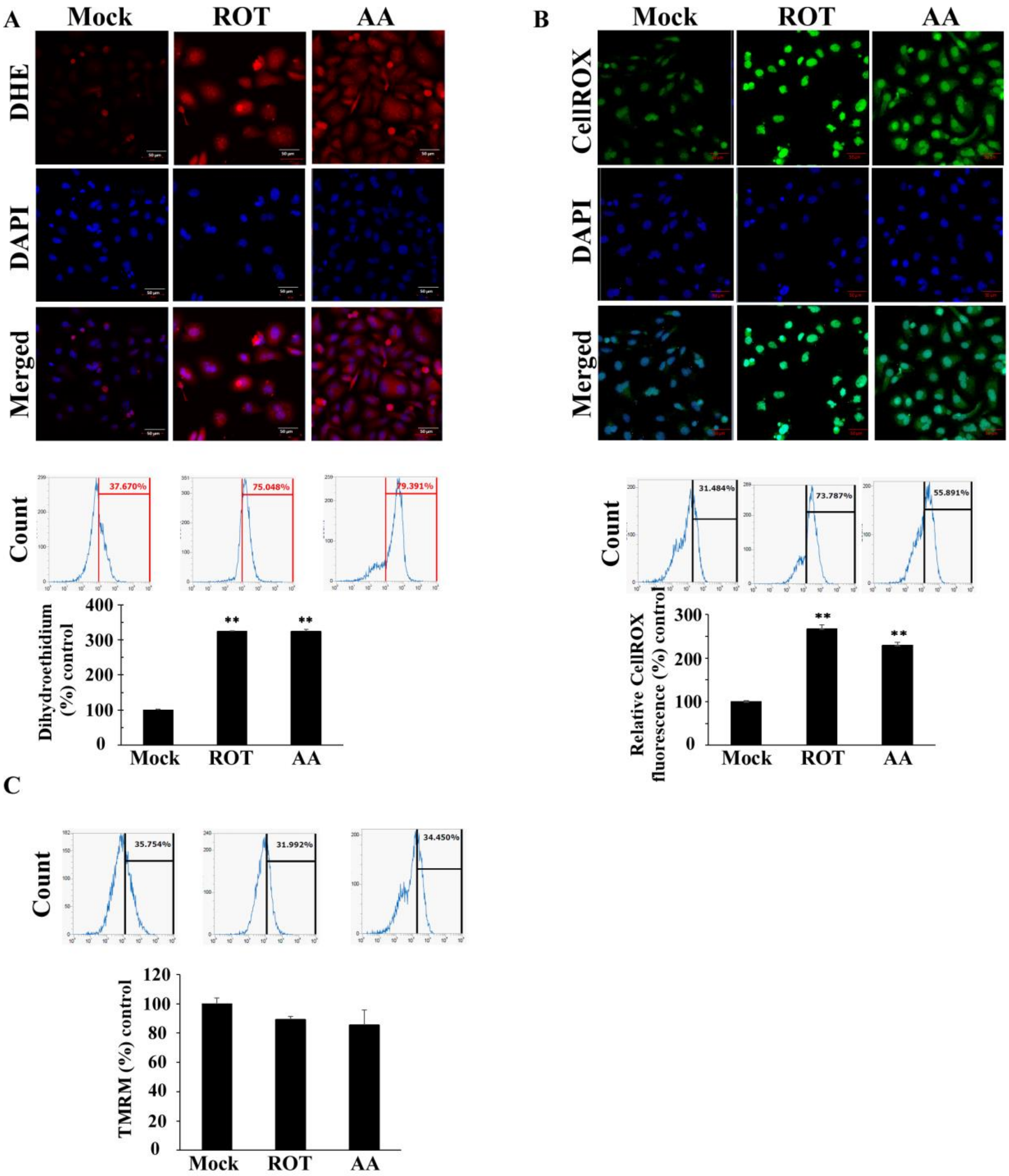

Supplement Figure 3

Supplementary Figure 3. The inhibitors of complex I (ROT) and complex III (AA) induced oxidative stress and decreased mitochondrial membrane potential (ΔΨm). (A, B, C) The mitochondrial ROS, cytosolic ROS and mitochondrial membrane potential (ΔΨm) levels were measured using flow cytometry and fluorescence microscopy with DHE Deep Red, CellROX Deep green and TMRM Deep Red staining, respectively, and indicated by the mean fluorescent intensity (MFI) or percentage of the cells.

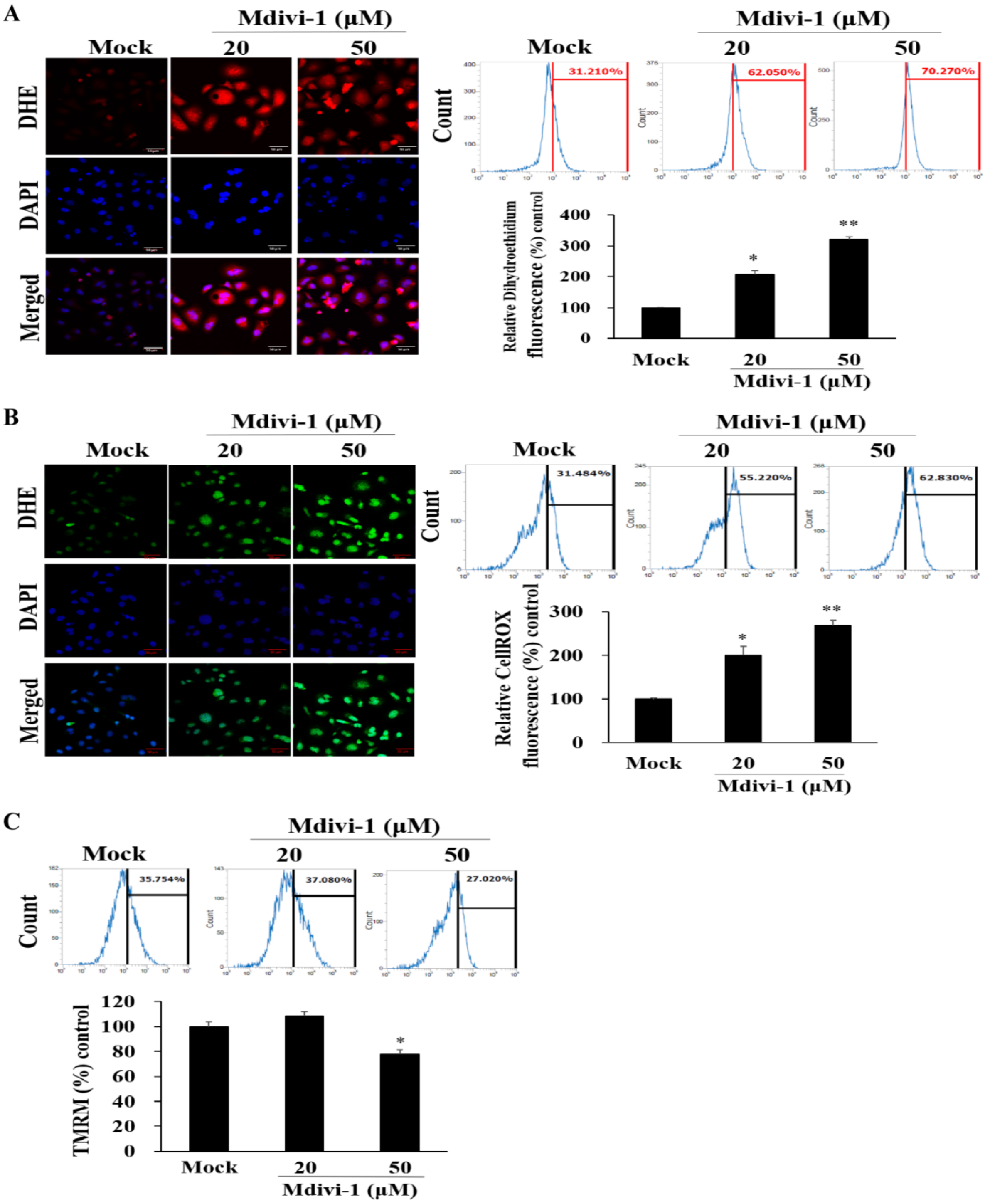

Supplement Figure 4

Supplementary Figure 4. Effect of mdivi-1 on the intracellular and mitochondrial ROS production in HeLa cells. (A, B, C) The mitochondrial ROS, cytosolic ROS and mitochondrial membrane potential (ΔΨm) levels were measured using flow cytometry and fluorescence microscopy with DHE Deep Red, CellROX Deep green and TMRM Deep Red staining, respectively, and indicated by the mean fluorescent intensity (MFI), or percentage of the cells.

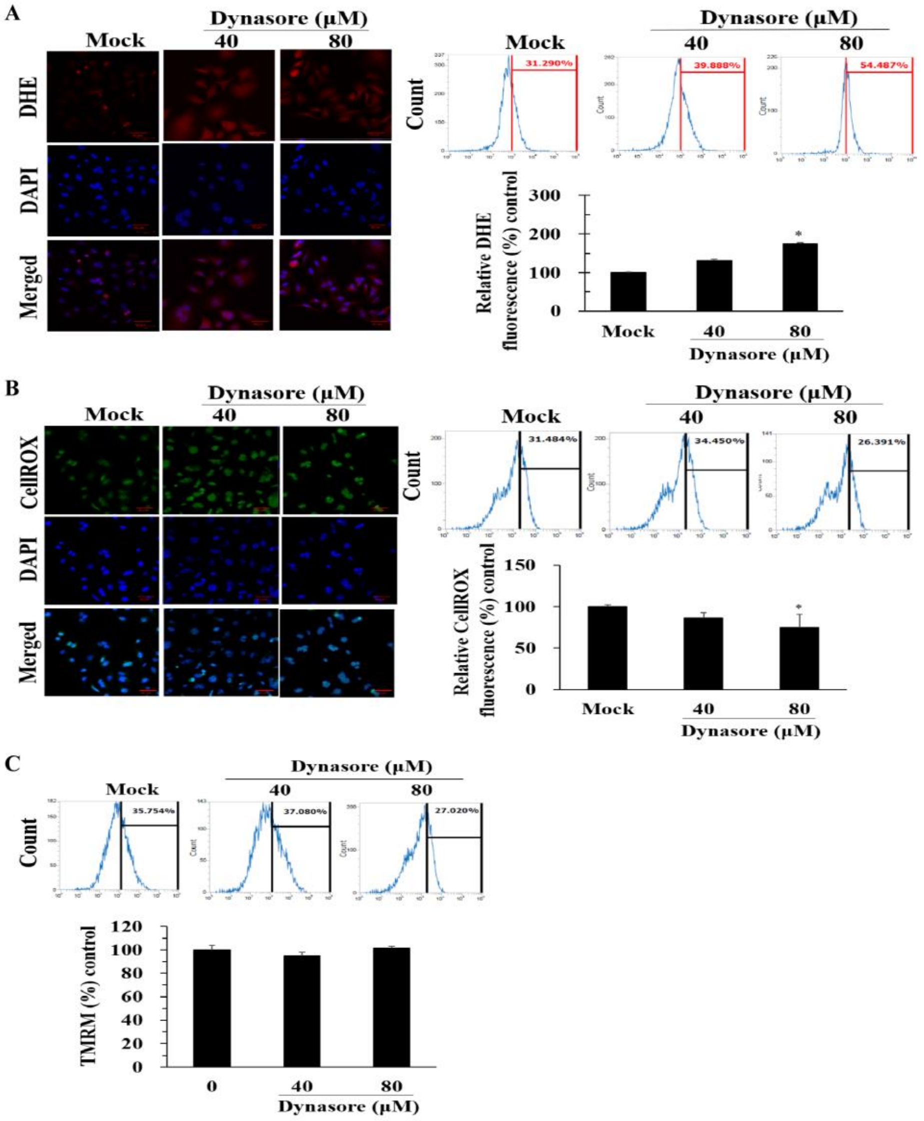

Supplement Figure 5

Supplementary Figure 5. Effect of mdivi-1 on the intracellular and mitochondrial ROS production in HeLa cells. (A, B, C) The mitochondrial ROS, cytosolic ROS and mitochondrial membrane potential (ΔΨm) levels were measured using flow cytometry and fluorescence microscopy with DHE Deep Red, CellROX Deep green and TMRM Deep Red staining, respectively, and indicated by the mean fluorescent intensity (MFI) or percentage of the cells.

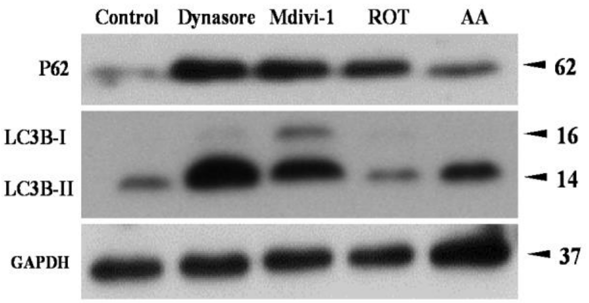

Supplement Figure 6

Supplementary Figure 6. LC3B-II/P62 immunoblotting was used to track the conversion of LC3B-I into LC3B-II and the expression of P62 for autophagic activity. HeLa cells were analyzed for centrosome amplification by treatment with 200 nM ROT, 10 μM AA, 50 μM Mdivi-1 and 80 μM Dynasore for 24 hrs. The lysates were subjected to western blotting and analyzed for P62 and LC3B-I/II.

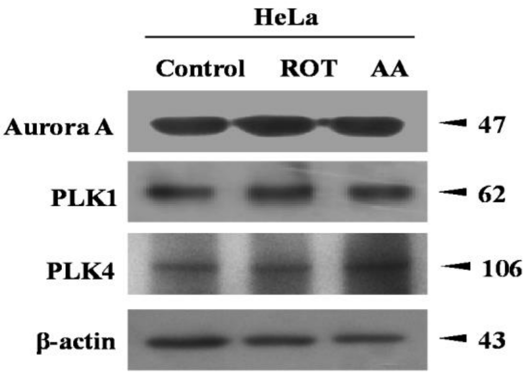

Supplement Figure 7

Supplementary Figure 7. Blocked mitochondrial ETC resulted in aberrant centrosomal and mitochondrial proteins in HeLa cells. HeLa cells were analyzed for centrosome amplification by treatment with 200 nM ROT or 10 μM AA for 24 hrs. The lysates were subjected to western blotting and analyzed for Aurora A, Plk1 and Plk4.

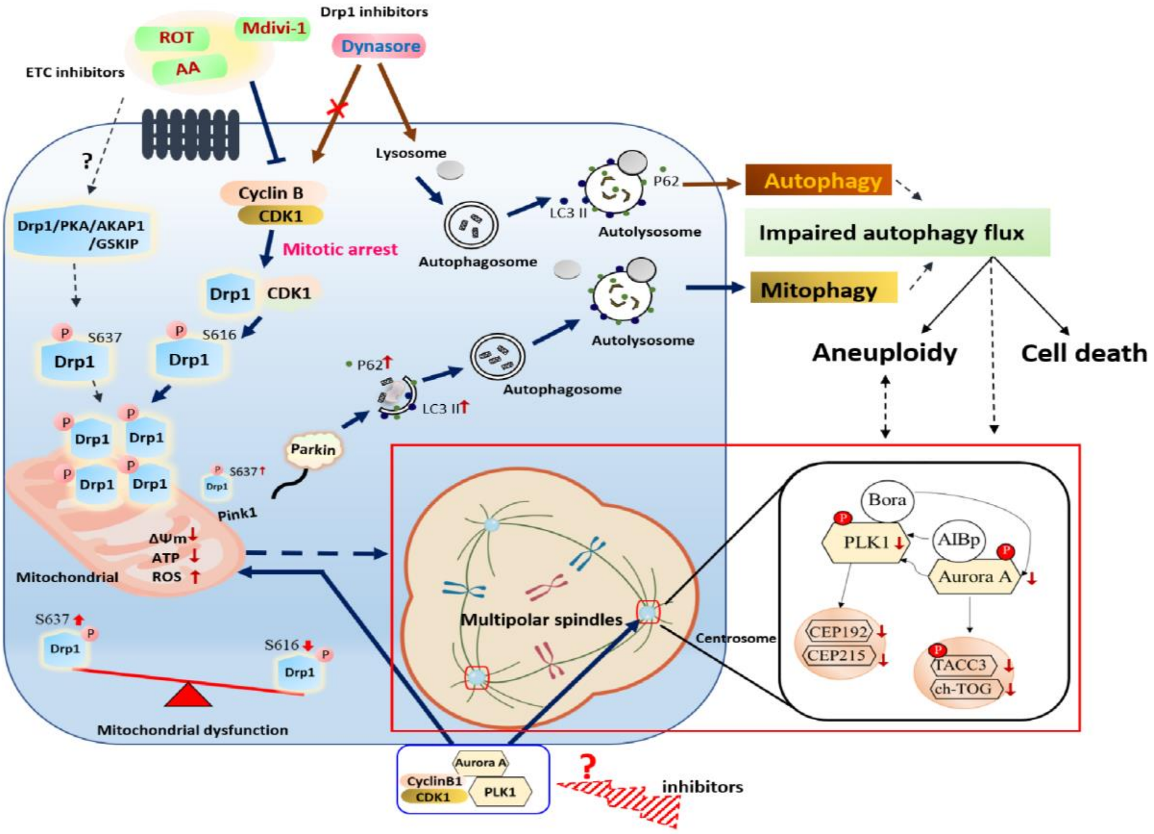

Supplement Figure 8

Supplementary Figure 8. Model showed that Drp1-Ser637 rather than Drp1-Ser616 via the PINK1/Parkin pathway results in membrane potential decrease, ATP production reduction, ROS increase and mitochondrial fragmentation. Ongoing mitophagy during mitotic arrest may simply represent disturbed M-phase kinases/proteins (Cdk1, Plk1 and Aurora A and their associated centrosomal proteins, Cyclin B, Bora, AIBp, CEP192/CEP215 and TACC3/chTOG) to induce multipolar spindles (labeled in red box). See the section of Discussion for details.
